# Why human lifespan is rapidly increasing: solving "longevity riddle" with "revealed-slow-aging" hypothesis

**DOI:** 10.18632/aging.100139

**Published:** 2010-04-18

**Authors:** Mikhail V. Blagosklonny

**Affiliations:** Department of Cell Stress Biology, Roswell Park Cancer Institute, BLSC, L3-312, Buffalo, NY 14263, USA

**Keywords:** aging, senescence, longevity, diseases, health span, lifespan, rapamycin, mTOR

## Abstract

Healthy life span is rapidly increasing and human aging seems to be postponed. As recently exclaimed in Nature, these findings are so perplexing that they can be dubbed the 'longevity riddle'. To explain current increase in longevity, I discuss that certain genetic variants such as hyper-active mTOR (mTarget of Rapamycin) may increase survival early in life at the expense of accelerated aging. In other words, robustness and fast aging may be associated and slow-aging individuals died prematurely in the past. Therefore, until recently, mostly fast-aging individuals managed to survive into old age. The progress of civilization (especially 60 years ago) allowed slow-aging individuals to survive until old age, emerging as healthy centenarians now. I discuss why slow aging is manifested as postponed (healthy) aging, why the rate of deterioration is independent from aging and also entertain hypothetical use of rapamycin in different eras as well as the future of human longevity.

## Unexpected increase in longevity

Death from aging is technically death from age-related diseases, which are manifestations of advanced aging [1]. But, historically, most people died young and, of course, not from age-related diseases but, rather, from starvation and epidemics (cholera, smallpox, tuberculosis and many currently rare infections) as well as from physical violence. Just three centuries ago, life expectancy was less than 16 years and 75% of people born in London in 1662 died before they reached the age of 26 (Graunt's life table). The progress of civilization eliminated many causes of death that killed young people in the past. This dramatically increased the average lifespan. In addition, modern medicine extended lifespan of old people by treating age-related diseases. But maximal lifespan seemed to be not affected. It was assumed that human life span is close to its upper limits. However, surprising demographists and gerontologists, it was shown that life expectancy continues to increase at an astonishing pace  [2,3]. In the countries with the highest life expectancies, the long term increase in life expectancy proceeds at a pace of 2.5 years per 10 years, or six hours per day [4]. A century ago, the chance to become centenarian (a person older than 100 years) was a hundred times lower. Furthermore, as calculated, most babies born since 2000 in countries with long life expectancies will celebrate their 100th birthdays [5]. Most astonishingly, people are reaching very old age in better health. But then they deteriorate fast, seemingly indicating that the rate of aging was not changed but just aging was postponed [3]. "Taken together, these findings are so perplexing that they can be dubbed the ‘longevity riddle': why do the evolutionary forces that shaped human aging provide a license to alter the level of health but not the rate of debilitation?" [3]. So why can aging be delayed but not slowed? Or can aging be slowed? In order to solve the longevity riddle, we should turn gerontology on its head. It has been always assumed that aging is caused by damage. As recently argued, aging is not driven by damage, but, in contrast, leads to damage (organ damage) [6-8]. And aging is driven in part by mTOR (mammalian target of rapamycin).

## TOR-driven quasi-programmed aging and age-related diseases

The mTOR intracellular signaling pathway is activated by numerous signals including glucose, amino acids, fat acids and other nutrients, insulin and some other hormones, growth factors and cytokines [9-11]. In response, it increases cellular functions and cellular mass growth [12]. When the cell cycle is blocked, mTOR drives cellular senescence [13]. Cellular aging can be defined as over-activation of signaling pathways (such as mTOR) with secondary signal resistance [14]. In turn this slowly leads to diseases of aging (hypertension, atherosclerosis, macular degeneration, insulin resistance, obesity, neurodegeneration, cancer, osteoporosis, organ hypertrophy). For example, TOR-dependent activation of osteoclasts causes bone resorption (osteoporosis) [15]. But these aging processes are relatively silent (subclinical, no obvious deterioration) until aging culminates in "catastrophes" - organ damage. For example, osteoporosis can lead to broken hip and atherosclerosis can lead to infarction. Then deterioration can be quick, leading to death in a mater of hours or years or decades, depending on the level of medical care.

## Morbid phase

When diseases become clinical then deterioration may be fast. For example, high blood pressure, thrombosis and atherosclerosis can culminate in stroke. This will initiate a chain of deteriorations (immobility - pneumonia, etc.) that are TOR-independent.  The duration of this morbid (deterioration) phase is almost solely determined by the level of medical care. Furthermore, age-related blindness and Alzheimer's disease are rarely lethal anymore. Medicine may dramatically prolong the morbidity phase, delaying death. Thus, the speed of deterioration is almost independent from the aging process and cannot serve as a marker of aging or the rate of aging. The rate of aging is actually determined by the age of the onset of age-related diseases. Slowing down the aging process (by calorie restriction, rapamycin or genetic manipulation) delays diseases.

## "Thought experiment": how would rapamycin affect longevity in 1667 versus 1967

Rapamycin is an anti-aging drug, which is currently used to prevent donor organ rejections [16].  Rapamycin delays cancer in animals and humans (see for review [17]). It also delays other age-related diseases in animal models of accelerated diseases. For example, rapamycin and its analogs delay atherosclerosis [18-23]. mTOR is involved in age-related diseases exactly because it is involved in aging. In fact, rapamycin prolongs life span in mice and flies [24-27]. It is expected that, in adult humans, rapamycin (at correct doses and schedules) will prolong healthy and maximal lifespan [16]. But consider rapamycin administered for life, starting from childhood. Then its effect on longevity will depend on the level of civilization and will be opposite in the 17^th^ and 20^th^ centuries.

**Scenario 1**. Assume that in 1667, 3 out of 4 newborns were randomly prescribed rapamycin for life. Rapamycin would slow down developmental growth (a disadvantage for survival, especially for orphans). Malnutrition and stresses would be less tolerated, because the nutrient sensing pathway is deactivated by rapamycin. Reduced muscle mass and fat stores would increase chances of death from violence and famine. In infants with natural immunotolerance, rapamycin would further decrease immunity against infections, which were numerous, incurable and non-preventable in 17^th^ century. So, if 3 out of 4 people must die before the age of 26 (1667 in London), they would be those who were treated with rapamycin. The control group would survive and develop diseases of aging at normal (early) age.

**Scenario 2**. In 20^th^ century London, sanitation, vaccination and other measures have greatly reduced epidemics. The discovery of antibiotics has further prevented death from infections. Famine and violent death are not common either. Those who were treated with rapamycin for life will survive into adulthood and then will age slowly.  In the rapamycin-treated group, diseases will be delayed. Furthermore, even its ability to cause immunologic tolerance (‘rejuvenate' immunity) will be beneficial in the elderly by decreasing hyper-immunity and autoimmunity. (Note: rapamycin improves immunity in old animals [28]).  So, now, the rapamycin treated group becomes centenarians in good heath. But because deterioration is mTOR-independent, this group will deteriorate at the same rate (but later in life) as the control group, assuming that the medical treatment is equal in both groups (in reality, younger patients are treated more intensively.)

## The revealed-slow-aging hypothesis

**Figure 1.**
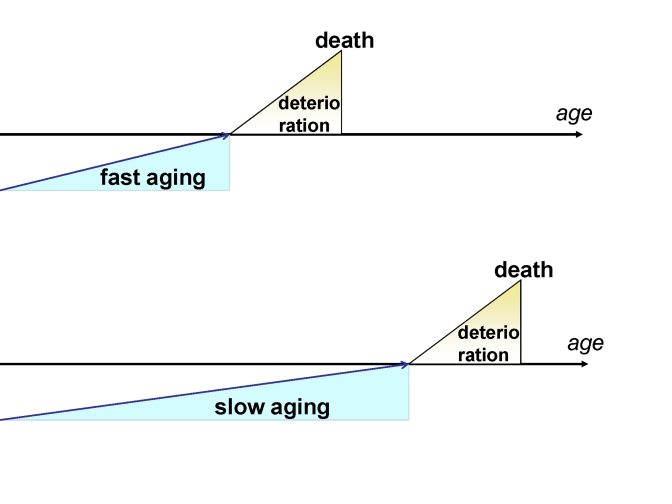
Fast and slow aging. In slow aging, the onset of deterioration is postponed but the rate of deterioration is not changed.

**Figure 2.**
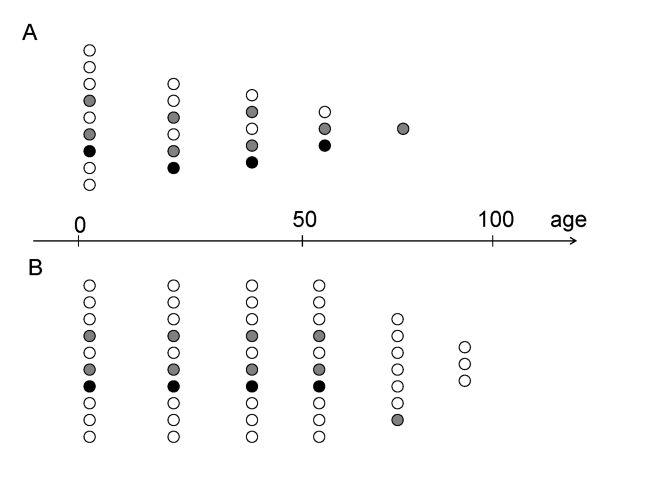
Preferential survival fast- versus slow-aging individuals. (**A**) In the past, slow-aging individuals (open circles) died prematurely and fast-aging individuals (closed circles) survived into old age. (**B**) Now, slow-aging individuals (open circles) survived into old age as healthy (biologically young) and outlive faster aging individuals (closed circles).

Thus, while slow aging was a disadvantage in 1667, it became an advantage in 1967. In the past, mostly fast-aging individuals could survive into chronologically old age (Figure 2A). Now, slow-aging individuals can survive into chronologically old age (Figure 2B). Therefore, demographists observe an increasing number of individuals who are healthy at advanced chronological ages with delayed onset of diseases, who then deteriorate at the same rate as younger patients (Figure 1A vs 1B).

Importantly, current increase in healthy lifespan (increased longevity with late onset of age-related diseases) is not caused by natural selection. It happens in the same generation. Slow aging was not selected but was simply revealed (Figure 2 B). Until recently, most slow-aging individuals died prematurely. They (we) did not necessarily die young but nevertheless died not from aging.  For example, at the same chronological age when fast-aging individuals died from heart attack, healthy slow-aging individuals died from malnutrition and infections, for instance. Elimination of premature death greatly enriched chronologically old population with slow-aging (biologically young) individuals (Figure 2).

To be possibly correct, the hypothesis requires a high proportion of slow-aging individuals at birth (Figure 2).  Otherwise, there would be too few slow-aging individuals to make a difference later (Figure 2 A vs B).  Why was not slow aging selected out? Slow aging must be beneficial for women, by increasing their reproductive period. In fact, female's fertility is decreasing early in life (starting from late twenties, long before menopause). This reproductive aging is one of the earliest manifestations of aging in females. So slow aging benefits females. Also, as I will discuss elsewhere, women do not need to be as robust as men, so can afford to age slower (see forthcoming article  "Why men age faster but reproduce longer: mTOR perspective"). In turn, males inherit genes for longevity too, explaining a high proportion of slow-aging individuals at birth.

The revealed-slow-aging hypothesis predicts that certain very harsh conditions may result in a decrease in healthy lifespan decades later. For example, perhaps it is robust (and therefore fast-aging later) young men who predominantly survived wars, camps and orphanages. (If so, the death of weak slow-aging young men during 1940^th^-1950^th^ might explain a drop in healthy lifespan of Russian men 50 years later.) Also, the hypothesis explains data on early-age mortality and subsequent mortality in the same cohorts. Thus Finch and Crimmins showed that increasing longevity and declining mortality in the elderly occurred among the same birth cohorts that experienced a reduction in mortality at younger ages [29,30]. The revealed-slow-aging hypothesis suggests that high levels of infection early in life eliminate young individuals with a ‘weak' mTOR (slow-aging individuals, who otherwise would live longer).

**Figure 3.**
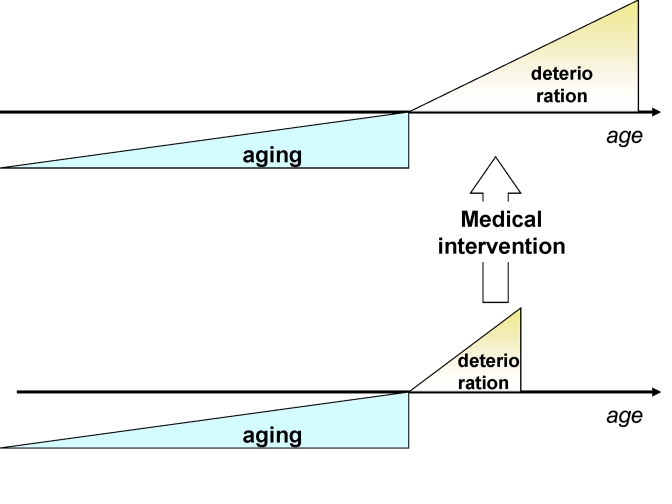
Traditional medicine increases survival (extends deterioration phase) without affecting the onset of deterioration.

## The prospect of longevity

Today, most slow-aging individuals, with less active mTOR, do not die early in life from malnutrition and infections and can reach chronologically old age. Exactly because they are slow-aging (young biologically), they are able to reach old age in good health. This may explain the current increase in longevity. But this trend is probably close to saturation and will be saturated by 2050 (a century after invention of antibiotics) in the countries with the highest longevity. The reason is that the rate of aging was not affected by elimination of death from famine and infections.

Yet, aging could be slowed by rapamycin, a drug currently approved to prevent organ rejection. (Note: rapamycin, as an anti-aging drug, perhaps should not be administrated until after growth is completed). Based on data with calorie restriction and rapamycin in mice, lifespan might be increased on 30 percent. Then we will observe 140-150 years old individuals and average lifespan will exceed 100.

## Solution of heath care crisis and further prospect on longevity

Currently, by treating each disease individually and focusing on advanced diseases, traditional medical interventions lengthen the morbidity phase (Figure 3).

**Figure 4.**
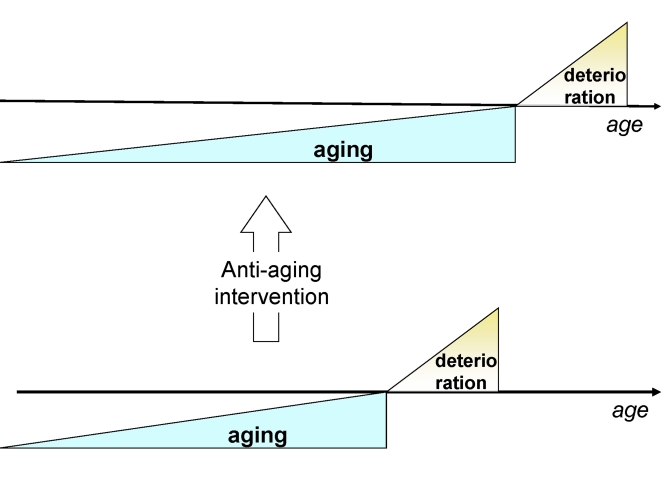
Anti-aging drugs will delay the onset of deterioration without affecting deterioration.

So, traditional medicine increases number of old people in bad health. However, extension of lifespan by lengthening only the morbidity phase will make the cost of medical care unsustainable for society. Anti-aging medicine can solve this crisis by delaying the morbidity (deterioration) phase (Figure 4).

There is incorrect perception that anti-aging drugs would increase a number of people suffering with age-related diseases. In contrast, such* old* people will be healthy because they will be only chronologically old but biologically young. They will be healthier for longer (until they reach biological age of deterioration). Biological age is by itself determined by the sum of all diseases of aging [1]. In other words, diseases of aging are manifestations of biological aging. It is impossible to dissociate biological aging and diseases of aging. Healthy aging is healthy *non-aging* (or slow aging).

Deceleration of aging, manifested as "healthy aging", increases the ratio of healthy to unhealthy people (Figure 4). Furthermore, the ability to work is determined by biological age. Slow aging may delay retirement until later in life (as also suggested by Vaupel [3]) and in turn may provide the means for society to support further development of increasingly powerful (and expensive) conventional medicine. Then lifespan can be extended by both anti-aging medical intervention (to delay morbidity) and specialized medical intervention (to prolong morbidity stage).

In conclusion, the progress of medicine 60-100 years ago (in prevention and treatment of non-age-related diseases) allowed slow-aging individuals to survive long enough to die from late onset age-related diseases (in other words to die from postponed aging). Civilization increased a proportion of slow-aging persons among the elderly, without actually slowing the aging process. Rapamycin will be used to slow down aging itself, further extending healthy lifespan. The extent of lifespan extension will depend on the future discoveries.  And future discoveries are predictably unpredictable [31].
